# Plasma Metabolic Features Associated with Developmental Regression in Autism Spectrum Disorder: A Pilot Untargeted Metabolomics Study

**DOI:** 10.3390/ijms27156995

**Published:** 2026-08-04

**Authors:** Cihan Aslan, Ozan Kaplan, Nadir Yalçın, Bilge Başak Fidan, Mustafa Çelebier, Dilek Ünal, Karel Allegaert

**Affiliations:** 1 Department of Child and Adolescent Psychiatry, Faculty of Medicine, Hacettepe University, Ankara 06230, Türkiye; cihan.aslan@hacettepe.edu.tr (C.A.); dilekunal@hacettepe.edu.tr (D.Ü.); 2 Department of Analytical Chemistry, Faculty of Pharmacy, Hacettepe University, Ankara 06100, Türkiye; ozankaplan@hacettepe.edu.tr (O.K.); celebier@hacettepe.edu.tr (M.Ç.); 3 Department of Clinical Pharmacy, Faculty of Pharmacy, Hacettepe University, Ankara 06100, Türkiye; 4 Department of Bioengineering, Graduate School of Science and Engineering, Hacettepe University, Ankara 06800, Türkiye; bilgefidan98@gmail.com; 5 Department of Development and Regeneration, KU Leuven, Leuven 3000, Belgium; karel.allegaert@kuleuven.be; 6 Department of Pharmaceutical and Pharmacological Sciences, KU Leuven, Leuven 3000, Belgium; 7 Department of Clinical Pharmacy, Erasmus Medical Center, Rotterdam 3015 GD, The Netherlands

**Keywords:** autism spectrum disorder, developmental regression, metabolomics, prostaglandin E3, sphingolipids, sialylation, Q-TOF LC/MS, biomarker

## Abstract

Developmental regression, the loss of previously acquired language and social skills during the second year of life, affects 20–40% of children with autism spectrum disorder (ASD), yet its metabolic underpinnings remain poorly defined. Existing metabolomics studies have focused on amino acid and acylcarnitine alterations, leaving the eicosanoid, sphingolipid, and glycoconjugate axes largely unexamined. Plasma from 25 children with regressive ASD (REG+), 27 with non-regressive ASD (REG−), and 21 typically developing controls (aged 2–6 years) was profiled using untargeted Q-TOF LC/MS (Agilent 6530, ESI+ mode, 100–1700 *m*/*z*). From 6657 detected features, a stepwise curation pipeline combining statistical significance (raw *p* < 0.05 with Benjamini–Hochberg FDR correction, *q* < 0.05; FC > 1.5), analytical plausibility, endogenous origin verification, and literature-based directional alignment yielded a prioritized set of 14 annotated metabolic features. Exploratory PLS-DA (Partial Least-Squares Discriminant Analysis) visualization showed separation among the three groups within the present dataset; however, diagnostic or predictive performance was not evaluated. The 14-feature panel clustered along four biochemical axes. A feature putatively annotated as prostaglandin E3 (PGE3) showed a 7.13-fold reduction in REG+ versus controls (1.58-fold in REG−; FC REG+/REG− = 4.52). N-Acetylneuraminosyl-(α2-6)lactosamine and aspartylglycosamine were decreased 4.44-fold and 5.42-fold in REG+, respectively, with regression-specific amplification (FC REG+/REG− > 2.0). Six sphingolipid–myelin metabolites showed coordinated biosynthetic depletion and catabolic elevation in both ASD groups without regression specificity. DHEA-S exhibited the largest subgroup differential (FC REG+/REG− = 8.89), with paradoxically greater depletion in the non-regressive group. This exploratory study identified several plasma metabolic features showing abundance differences among children with REG+, REG−, and typically developing controls. These findings are hypothesis-generating and require confirmation using authenticated standards, targeted quantitative methods, and independently recruited cohorts before any diagnostic, predictive, or therapeutic relevance can be established.

## 1. Introduction

Autism spectrum disorder (ASD) is a neurodevelopmental condition defined by persistent difficulties in social communication and interaction, alongside restricted and repetitive patterns of behavior, interests, or activities. Developmental regression, defined as the loss of previously acquired skills, most commonly in language and social communication, occurs in an estimated 20–40% of children later diagnosed with ASD [1,2]. The disorder affects an estimated 1 in 36 children in the United States and carries a comparable burden worldwide, yet its etiology remains incompletely understood. Although individuals with ASD share core diagnostic features, the clinical presentation is remarkably heterogeneous across cognitive ability, language development, co-occurring medical conditions, and developmental trajectory [2,3]. The timing of regression, typically during the second year of life, coincides with peak synaptogenesis and myelination in the developing brain [3,4,5], a period of peak metabolic demand in the central nervous system [4]. Despite its frequency, the causes of regression remain unknown; candidate factors such as epilepsy, epileptiform electroencephalographic patterns, and early childhood immunizations have not been supported as causal or even correlational triggers [5]. The majority of ASD diagnoses are still made at age three or later, well after the window in which regression typically occurs, underscoring the urgent need for objective biological markers that could enable earlier identification of at-risk children [3,5].

Metabolomics, the systematic profiling of small-molecule metabolites in biological fluids and tissues, is increasingly used to characterize the biochemical features of ASD. Unlike genomics or transcriptomics, metabolomics captures the downstream consequences of gene–environment interactions in real time, making it particularly suited to disorders in which environmental and physiological stressors modulate a genetically predisposed background [6,7,8]. Over the past decade, a growing body of plasma, urine, and cerebrospinal fluid metabolomics studies has identified consistent perturbations in amino acid metabolism, purine turnover, oxidative stress markers, and mitochondrial bioenergetics in ASD cohorts [4,5,6,7]. A smaller subset of studies has specifically compared children with regressive (REG+) versus non-regressive ASD (REG−). Rangel-Huerta et al. (2019) employed the Metabolon Global Metabolomics platform encompassing four orthogonal UPLC-MS/MS methods and methanol-based protein precipitation on plasma from 30 ASD children (15 with regression) and 30 controls, reporting significant alterations in nicotinamide metabolism, energy metabolism, and select amino acid pathways [4]. Priyanka et al. (2025) applied a targeted MS/MS panel covering 52 metabolites (primarily amino acids and acylcarnitines) in 70 ASD and 58 control children, identifying acylcarnitine species and adenosine as leading biomarker candidates with a Random Forest classification accuracy of 79.5% [5]. These studies advanced the field considerably; however, their analytical designs were not optimized for eicosanoid or sphingolipid detection. The Rangel-Huerta panel, despite its breadth, did not return any prostaglandin or sphingolipid species among its significantly altered metabolites. The Priyanka panel excluded both classes by design, focusing instead on a clinical NeoBase-type assay. Similarly, El-Ansary et al. (2021) characterized the prostaglandin axis in ASD plasma through sandwich ELISA and reported a 140% elevation in cyclooxygenase-2 and a 124% elevation in prostaglandin E2-EP2 receptor levels, but did not measure any omega-3-derived prostaglandin species [9]. In aggregate, the existing literature provides detailed coverage of amino acid, acylcarnitine, and pro-inflammatory eicosanoid alterations, while leaving the anti-inflammatory and resolution arms of lipid metabolism, sphingolipid dynamics, and glycoconjugate biology largely unexamined in the context of autistic regression.

Three areas of metabolism that are directly relevant to neurodevelopment have received almost no attention in ASD regression research. First, prostaglandin E3 (PGE3), the principal cyclooxygenase product of eicosapentaenoic acid (EPA), attenuates COX-2 gene expression and limits interleukin-6 secretion [10], while opposing PGE2 signaling through differential receptor engagement [11]. Although PGE2 elevation has been documented in ASD [9], PGE3 levels have not been reported in ASD populations. Second, sialylated glycoconjugates, in particular those carrying α2,6-linked N-acetylneuraminic acid, are critical for neural cell adhesion molecule function, synaptic plasticity, and axon guidance during early brain development [12,13]. Altered total sialic acid levels have been reported in ASD plasma and saliva through colorimetric assays [14,15], and sialic acid supplementation improved social behavior in a valproic acid autism model [16]; however, intact sialylated metabolites have not been reported in untargeted ASD metabolomics studies, possibly because the gas chromatography–mass spectrometry (GC-MS) platforms used in earlier studies are not well suited to thermally labile sialic acid linkages [17,18]. Third, sphingolipid metabolism, which governs myelination, membrane microdomain organization, and blood–brain barrier integrity, has recently been implicated in autistic regression through cerebrospinal fluid analyses showing the dysregulation of 21 out of 40 measured sphingolipid species [19,20]. Whether these central nervous system sphingolipid disturbances are reflected in peripheral blood—and whether they differ between REG+ and REG− has not been determined. The marked clinical heterogeneity of ASD is thought to be mirrored at the molecular level, where distinct metabolic patterns may correspond to specific clinical phenotypes. In this context, developmental regression represents a particularly informative subtype for metabolomic stratification [7].

The present pilot study was designed to address these gaps. Using quadrupole time-of-flight liquid chromatography–mass spectrometry (Q-TOF LC/MS) in positive electrospray ionization mode with a broad scanning range of 100–1700 *m/z*, we profiled the plasma metabolome of 25 children with REG+, 27 children with REG−, and 21 healthy controls recruited at a tertiary referral care university hospital.

The untargeted design and the mass accuracy of the Q-TOF instrument (≤5 ppm) allowed for the simultaneous detection of eicosanoids, sphingolipids, and glycoconjugates within a single analytical run—metabolite classes that previous ASD metabolomics platforms either excluded or could not resolve. The study was designed as a discovery-phase metabolomics investigation intended to identify biologically informative plasma signals associated with developmental regression in ASD rather than to construct a definitive diagnostic classifier. Our primary objectives were (i) to characterize plasma metabolic features showing abundance differences among children with REG+, REG−, and typically developing controls; (ii) to explore eicosanoid-, sphingolipid-, and sialylated glycan-related features potentially associated with developmental regression in ASD; and (iii) to interpret these exploratory findings within the broader pathophysiological literature. The study was not designed to construct or validate a diagnostic classifier or to estimate sensitivity, specificity, or predictive performance.

## 2. Results

### 2.1. Baseline Demographic and Clinical Characteristics

Baseline demographic and clinical variables did not differ significantly across groups (all *p* > 0.05). Age, sex distribution, anthropometric measures (weight, height, BMI), and CARS scores were comparable across the three groups. CARS scores did not differ between the REG+ and REG− groups. Mean age ranged from 40.5 ± 14 to 43.14 ± 12 months across groups, with similar female representation (16–19%). CARS scores did not differ between REG+ (45.8 ± 3.7) and REG− (46.9 ± 5.5), indicating comparable clinical severity across ASD subgroups.

### 2.2. Global Metabolomic Profiling and Multivariate Separation

The untargeted Q-TOF LC/MS analysis of plasma from the three study groups (REG+, *n* = 25; REG−, *n* = 27; C, *n* = 21) detected 6657 chromatographic features. Following initial statistical filtering based on raw *p* < 0.05 and FC > 1.5, Benjamini–Hochberg FDR correction and the stepwise curation procedure detailed in Section 4.5 were applied. A final panel of 14 prioritized endogenous metabolic features that remained significant at q < 0.05 in at least one comparison was retained (Table 1). Complete analytical and statistical results for all 68 annotated chromatographic features, including raw *p*-values, Benjamini–Hochberg-adjusted q-values, fold-change values, retention times, mass errors, and metabolite identification confidence, are provided in Supplementary Table S1 and S2.

**Figure 1:**
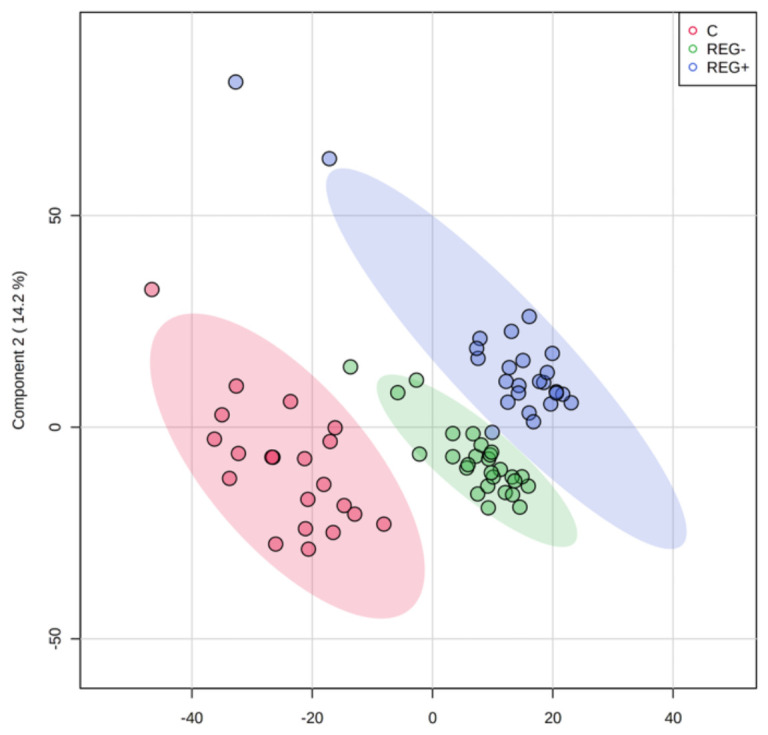
PLS-DA score plot of the three study groups (C: red, REG−: green, REG+: blue).

**Figure 2:**
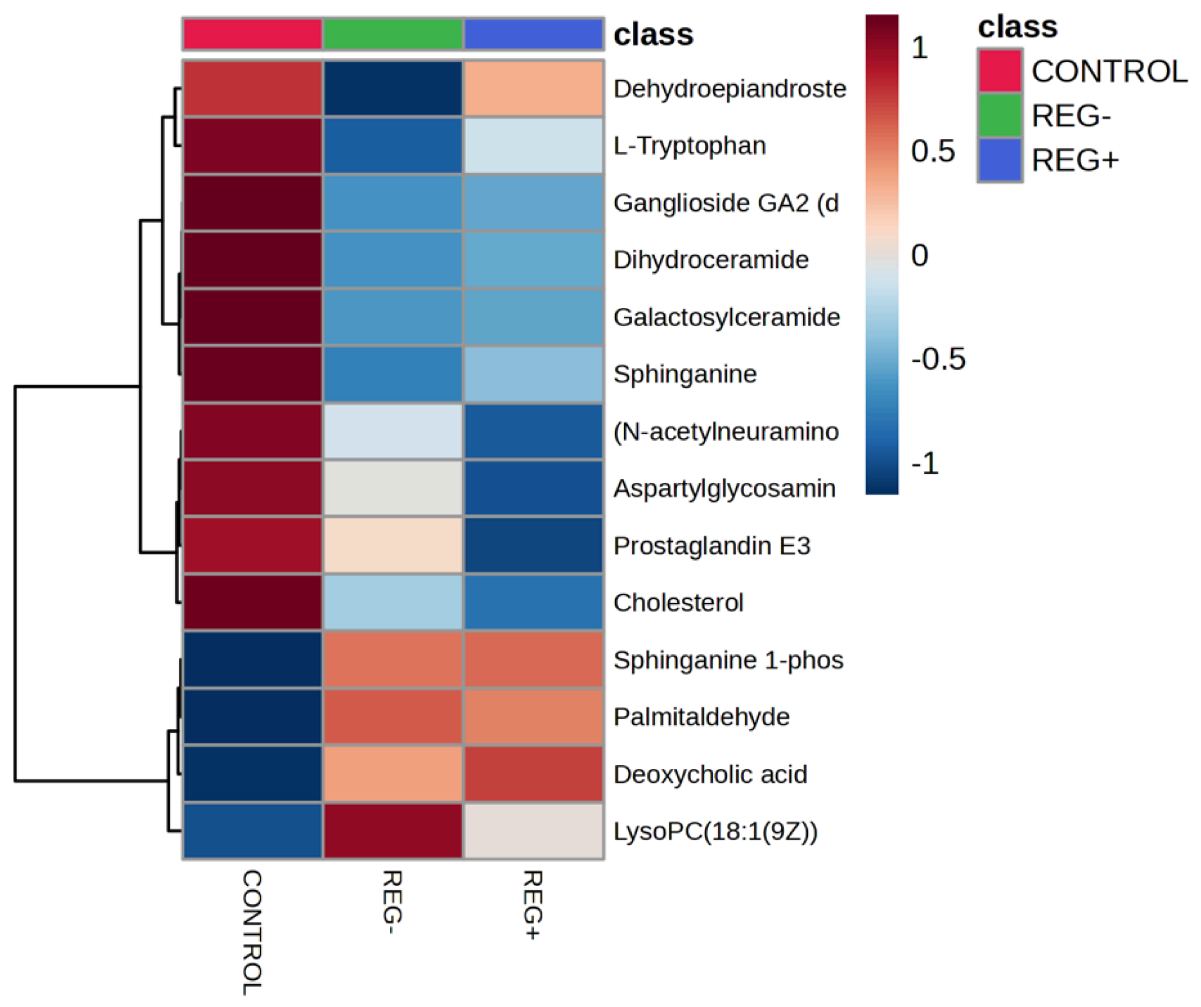
Heatmap illustrating the relative abundance of prioritized metabolic features among the C, REG−, and REG+ groups, with red indicating higher levels and blue indicating lower levels. The metabolite profile showed group-associated relative abundance patterns within the present cohort, demonstrating a gradient of metabolic alteration from controls through REG− to REG+.

Four prioritized metabolic features showed >2-fold numerical abundance differences between the REG+ and REG− groups and remained significant after Benjamini–Hochberg correction in the REG+ vs. REG− comparison: PGE3 (q = 4.37 × 10^−3^), DHEA-S (q = 5.61 × 10^−4^), aspartylglycosamine (q = 5.32 × 10^−3^), and N-acetylneuraminosyl-(α2-6)lactosamine (q = 9.28 × 10^−3^). Exploratory PLS-DA visualization showed apparent separation among the three groups within the present dataset (Figure 1). The heat map demonstrated group-associated relative abundance patterns across the prioritized metabolic features (Figure 2). Because these analyses were exploratory and no independent validation was performed, the observed patterns should not be interpreted as evidence of diagnostic or classification performance.

The 14 metabolic features span four biochemical domains. Individual results are presented below under these groupings: (i) sphingolipid–myelin axis, (ii) inflammation resolution pathway, (iii) glycosylation and sialylation axis, and (iv) gut–brain axis and systemic metabolism.

### 2.3. Sphingolipid–Myelin Axis

Six sphingolipid–myelin metabolites were altered in ASD plasma relative to controls (Table 1). Sphinganine 1-phosphate was elevated 2.40-fold in REG+ and 2.36-fold in REG−; the two ASD groups did not differ (FC REG+/REG− = 1.02). Sphinganine was decreased 1.54-fold in REG+ and 1.73-fold in REG−.

Dihydroceramide was decreased 4.16-fold in REG+ and 5.22-fold in REG−. Galactosylceramide (d18:1/16:0) was decreased 3.53-fold in REG+ and 3.91-fold in REG−. Ganglioside GA2 (d18:1/12:0) was decreased 2.00-fold in REG+ and 2.13-fold in REG−. None differed between ASD subgroups (FC REG+/REG− = 1.06–1.25). Palmitaldehyde was elevated 1.70-fold in REG+ and 1.76-fold in REG−.

### 2.4. Inflammation Resolution Pathway

The feature annotated as PGE3 was decreased 7.13-fold in REG+ relative to controls. In REG−, PGE3 was decreased 1.58—a markedly smaller reduction. The FC REG+/REG− ratio was 4.52, representing the second-largest numerical fold-change difference between the ASD subgroups after DHEA-S. LysoPC(18:1(9Z)) was elevated 1.26-fold in REG+ and 1.53-fold in REG− (FC REG+/REG− = 1.21).

### 2.5. Glycosylation and Sialylation Axis

The feature annotated as N-acetylneuraminosyl-(α2-6)lactosamine was decreased 4.44-fold in REG+ and 1.83-fold in REG− (FC REG+/REG− = 2.43). Aspartylglycosamine was decreased 5.42-fold in REG+ and 1.75-fold in REG− (FC REG+/REG− = 3.09). Both followed the same pattern: modest decreases across the broader ASD spectrum, with amplified reductions in the regressive subgroup.

### 2.6. Gut–Brain Axis and Systemic Metabolism

Deoxycholic acid was elevated 6.97-fold in REG+ and 5.84-fold in REG− (FC REG+/REG− = 1.19). L-Tryptophan was decreased 1.35-fold in REG+ and 1.76-fold in REG−. Cholesterol was decreased 2.37-fold in REG+ and 1.73-fold in REG−.

DHEA-S displayed a distinct profile: decreased 1.94-fold in REG+ and 17.23-fold in REG−. DHEA-S showed the largest numerical fold-change difference between the ASD subgroups (FC REG+/REG− = 8.89). Unlike all other panel metabolites, DHEA-S was more severely reduced in the non-regressive group.

Four prioritized metabolic features—PGE3 (FC REG+/REG− = 4.52), DHEA-S (8.89), aspartylglycosamine (3.09), and N-acetylneuraminosyl-(α2-6)lactosamine (2.43)—exhibited fold-change ratios exceeding 2.0 between the two ASD subgroups. The remaining ten were altered in both subgroups without a marked regression-specific gradient.

## 3. Discussion

Four of the 14 prioritized metabolic features—PGE3, N-acetylneuraminosyl-(α2-6)lactosamine, aspartylglycosamine, and DHEA-S—showed >2-fold numerical abundance differences between the REG+ and REG− groups and remained significant after FDR correction, with q-values of 4.37 × 10^−3^, 9.28 × 10^−3^, 5.32 × 10^−3^, and 5.61 × 10^−4^, respectively. These findings indicate statistically supported group-associated abundance differences, but do not establish that the features can discriminate between ASD phenotypes at the individual level. The results should therefore be regarded as exploratory findings requiring targeted confirmation and independent replication. The remaining ten were altered in both subgroups with comparable magnitude when compared to control children. The discussion therefore first considers the subgroup-specific findings and then the metabolic alterations shared by both ASD subgroups. Importantly, current evidence suggests that no single metabolite is sufficient to serve as a reliable biomarker in ASD; rather, panels of metabolites reflecting coordinated pathway-level alterations are more likely to capture the underlying biological heterogeneity [7].

Previous metabolomics studies comparing REG+ and REG− have generally reported modest subgroup differences, often limited to specific metabolic pathways rather than large-scale separation [7]. In this context, the relatively pronounced subgroup differences observed in the present study should be interpreted cautiously and require independent replication.

### 3.1. Inflammation-Related Findings: The Putative PGE3 Signal

The feature putatively annotated as PGE3 was reduced 7.13-fold in REG+ plasma relative to controls—the largest single-metabolite change in the dataset—while REG− showed only a 1.58-fold reduction. The REG+/REG− fold-change ratio was 4.52, the second-largest subgroup difference after DHEA-S. PGE3 opposes PGE2 at multiple levels: it induces less than half of the COX-2 transcription, generates substantially less IL-6 in macrophages [10], and acts as a partial antagonist at EP3 and EP4 receptors [21]. Its near-total loss in the REG+ group raises the possibility that the neuroinflammatory process documented in ASD [9] proceeds without adequate counter-regulation, suggesting compromised resolution capacity rather than an excess of initiation alone.

A possible enzymatic explanation for this finding has been described. Laneuville et al. demonstrated, through kcat/Km measurements, that human COX-2 oxygenates arachidonic acid with far greater catalytic efficiency than EPA [22]. When both fatty acids compete for the same enzyme, allosteric cross-talk between COX-2 homodimer subunits further amplifies this selectivity: EPA occupies the allosteric monomer, causing the partner catalytic site to preferentially process AA [23]. In our cohort, COX-2 is presumably upregulated (140% elevation reported by El-Ansary et al. [9]) and phospholipase A_2_ activity is increased—reflected by elevated LysoPC(18:1) in both ASD groups—liberating AA from membrane stores. Under these conditions, COX-2 metabolism may favor PGE2 over PGE3. The combination of elevated LysoPC and reduced PGE3 is consistent with this proposed substrate shift.

Previous omega-3 intervention studies in ASD have produced mixed results [24]. In this context, the observed PGE3-related pattern provides a hypothesis for future studies examining the relationships among EPA availability, prostaglandin metabolism, and inflammation-related pathways in ASD. However, the present untargeted and cross-sectional data do not establish PGE3 as a pharmacodynamic endpoint or support a specific therapeutic intervention. Confirmation of metabolite identity, targeted quantification, and prospective validation would be required before any interventional relevance could be considered.

### 3.2. Glycosylation- and Sialylation-Related Findings

N-Acetylneuraminosyl-(α2-6)lactosamine was depleted 4.44-fold in REG+ compared with 1.83-fold in REG− (REG+/REG− = 2.43). Aspartylglycosamine followed the same pattern: 5.42-fold in REG+ versus 1.75-fold in REG− (REG+/REG− = 3.09). These two metabolites occupy opposite ends of glycoprotein metabolism—surface sialylation and lysosomal turnover—yet their co-directional, regression-specific collapse points to a broad impairment of N-linked glycan biology.

The polysialylation of NCAM governs neuronal migration and circuit plasticity, and polysialyltransferase gene polymorphisms (*ST8SIA2*, *ST8SIA4*) are associated with ASD [12,13]. The α2,6 linkage modulates integrin-mediated adhesion and immune cell trafficking [12], connecting this axis to the inflammation resolution deficit. The parallel decline of aspartylglycosamine—whose accumulation in aspartylglucosaminuria causes progressive intellectual disability [25]—adds a degradative dimension. Ganglioside GA2 bridges both sphingolipid and glycosylation domains.

Preclinical findings indicating that sialic acid supplementation improved behavioral and metabolic outcomes in a valproic acid model of autism provide biological context for the observed glycosylation-related patterns [16]. However, these preclinical findings do not establish the clinical relevance or therapeutic applicability of sialic acid supplementation in children with regressive ASD. Future studies should first confirm the observed plasma features and clarify their relationship with glycan biology.

### 3.3. Systemic Metabolic Differences: DHEA-S and the Gut–Brain Axis

DHEA-S showed the largest numerical abundance difference between the ASD subgroups (REG+/REG− = 8.89), but its direction was inverted: depleted 17.23-fold in REG− but only 1.94-fold in REG+. Croonenberghs et al. reported an elevated cortisol-to-DHEA-S ratio in autistic individuals, interpreting the imbalance as a shift toward neurotoxic adrenal output [26], and Strous et al. independently confirmed lowered DHEA-S in adults with the disorder [27]. Our data complicate this picture by revealing that DHEA-S depletion diverges sharply between subtypes. One reading is that the non-regressive group has exhausted its DHEA-S reserve through sustained HPA axis demand, whereas the regressive group retains a relatively higher pool, possibly reflecting a distinct adrenal maturation trajectory that fails to prevent regression because downstream targets (sialylation, inflammation resolution) are already compromised.

Deoxycholic acid, the most elevated metabolite in both groups (6.97-fold in REG+, 5.84-fold in REG−), provides plasma-level evidence for gut dysbiosis [28]. This secondary bile acid is produced by colonic *Clostridium* spp. [29], and microbiota transfer therapy normalizes bile acid-related plasma metabolites in ASD [30]. Tryptophan depletion (~1.5-fold) and cholesterol depletion (2.37-fold in REG+, 1.73-fold in REG−) are among the most replicated ASD metabolomic findings [31,32,33]; their presence confirms that the cohort displays the canonical ASD metabolic signature.

Consistent with previous studies of regressive ASD, subgroup-related metabolic differences in the present dataset were observed across lipid metabolism, amino acid pathways, and gut-related metabolites, supporting the notion that regression may be associated with system-level metabolic alterations rather than isolated pathway disruptions [7].

### 3.4. Shared Metabolic Landscape: The Sphingolipid–Myelin Axis

Six sphingolipid–myelin axis metabolites were altered with comparable magnitude in both ASD subgroups (all REG+/REG− ≤1.25), positioning this pathway as a shared vulnerability rather than a regression trigger compared to the findings in control children. Sphinganine was reduced ~1.6-fold, while dhS1P was elevated ~2.4-fold, implicating upregulated sphingosine kinase activity diverting precursor from ceramide synthesis. Dihydroceramide fell ~4.7-fold, galactosylceramide ~3.7-fold, and ganglioside GA2 ~2.1-fold. Palmitaldehyde rose ~1.7-fold, confirming irreversible catabolism of the sphingoid backbone.

Yan et al. reported a parallel CSF sphingolipid signature in autistic regression, attributed to acid sphingomyelinase activation [19]. Our plasma data replicate that pattern in peripheral blood and demonstrate that it is not confined to the regressive subtype. Circulating dhS1P modulates blood–brain barrier permeability through S1P receptor signaling [34], and S1PR1 antagonism in BTBR autism mice restored neurovascular integrity [35]. White matter abnormalities are among the most consistent neuroimaging findings in ASD [36]; the combined depletion of galactosylceramide and cholesterol offers a candidate biochemical mechanism.

These observations are also consistent with the recent systematic review by Srivastava and O’Reilly, which showed that CSF alterations in ASD are not restricted to sphingolipid metabolism but extend to immune mediators, proteins, folate-related parameters, oxytocin, vasopressin, and changes in CSF physiology [37]. The available evidence, however, remains heterogeneous with respect to study populations, analytical methods, and the CSF markers examined. Moreover, because paired plasma and CSF samples were not collected in the present study, the metabolomic findings detected in plasma cannot be assumed to directly reflect CSF metabolite levels or biochemical processes. This comparison is therefore presented to provide biological context for the observed peripheral metabolic changes and should not be interpreted as evidence that these changes originate from, or directly represent, the central nervous system.

Our results converge on a two-tier model. The first tier—sphingolipid–myelin depletion, bile acid excess, tryptophan and cholesterol deficiency—is shared across the autism spectrum and may establish a vulnerable neural substrate. The second tier—PGE3 collapse, sialylation failure, and the distinctive DHEA-S divergence—is specific to or amplified in the regressive phenotype. Whether the shared tier precedes and enables the regression-specific insults cannot be resolved from cross-sectional data. Longitudinal metabolomic profiling before the typical age of regression onset will be essential to test this model.

### 3.5. Limitations

Several methodological constraints should be considered when interpreting these findings. First, the untargeted design, while enabling broad metabolome coverage, yields putative (MSI Level 2) identifications based on accurate mass, isotope pattern, and chromatographic behavior rather than co-elution with authenticated reference standards. In addition, no MS/MS data were acquired; therefore, putative annotations could not be supported by diagnostic product-ion spectra, and isomeric or isobaric candidates could not always be distinguished. The metabolite annotations reported here require confirmation through targeted assays employing authentic reference standards and MS/MS spectral matching in well-phenotyped ASD cases before any diagnostic application can be considered.

Second, the cohort size (25 REG+, 27 REG−, 21 controls) is appropriate for a discovery-phase pilot study, but limits statistical power for subgroup analyses and does not permit adjustment for multiple demographic or clinical confounders—such as sex, age, gastrointestinal comorbidities, sleep patterns, dietary variability, and unmeasured environmental exposures—that may influence plasma metabolite levels. Replication in larger, independently recruited, and clinically well-characterized multi-center cohorts is a prerequisite for establishing the reproducibility and generalizability of the reported metabolic signatures.

Third, the cross-sectional design captures a single time point and cannot establish temporal precedence. Whether the regression-specific metabolite alterations (PGE3, sialylated glycans, DHEA-S) precede, accompany, or follow the onset of skill loss remains unknown. Longitudinal sampling initiated before the typical regression window (12–24 months) in at-risk infants would be required to address causality. Finally, the study employed positive-mode ESI exclusively; metabolites that ionize preferentially in negative mode may have been missed, and the addition of dual-polarity acquisition in future work would extend metabolome coverage.

In addition, the feature-selection and panel-prioritization strategy was exploratory and biologically informed and therefore may be vulnerable to selection bias inherent to discovery-phase untargeted metabolomics. Similarly, supervised multivariate visualization methods such as PLS-DA may overstate group separation in modestly sized cohorts if not independently validated. Accordingly, the present findings should be interpreted as hypothesis-generating until replicated with targeted quantification and external validation.

The present study did not include ROC/AUC analysis or an independently recruited validation cohort and therefore cannot establish sensitivity, specificity, predictive value, or diagnostic performance for any individual metabolic feature or multifeature combination. Because exploratory feature prioritization was performed using the same dataset, post hoc ROC analysis in this cohort could yield optimistically biased performance estimates. Targeted confirmation using authenticated standards, prespecified analytical models, and independent validation in larger cohorts will be required before any clinical relevance can be assessed.

## 4. Materials and Methods

### 4.1. Study Population and Clinical Recruitment

This cross-sectional case–control study was conducted at Hacettepe University, Ankara, Türkiye. The study population comprised three groups: children diagnosed with regressive ASD (REG+, *n* = 25), children diagnosed with non-regressive ASD (REG−, *n* = 27), and typically developing healthy controls (*n* = 21), comparable in age and sex distribution to the ASD groups. All participants were between 2 and 6 years of age at the time of enrolment and were recruited between January 2020 and January 2022. ASD diagnosis was established by experienced child and adolescent psychiatrists according to DSM-5 criteria [38]. To support diagnostic confirmation and assess symptom severity, the Childhood Autism Rating Scale (CARS) was administered to all participants with ASD.

Regression status was determined through detailed clinical history obtained from parents or primary caregivers during psychiatric assessment and corroborated, where available, by medical records documenting early developmental milestones and subsequent skill loss. Classification was based on clinician consensus rather than a standalone regression-specific instrument. When available, retrospective home video recordings were also reviewed to support parental reports and provide additional context regarding early developmental trajectories.

Classification into regressive and non-regressive subgroups was based on a documented history of developmental regression, defined as a clinically significant loss of previously acquired skills in both language and social communication domains, following a period of apparently typical or near-typical early development. Consistent with established definitions, regression was operationalized as a loss of expressive language (e.g., loss of meaningful words or communicative intent) accompanied by a concurrent decline in social engagement (e.g., reduced eye contact, diminished joint attention, or decreased social reciprocity), rather than a simple developmental plateau. To ensure a homogeneous and clinically robust regressive phenotype, only children demonstrating loss in both domains were included in the regression group. The loss was required to persist for at least three months and to occur before 36 months of age, in line with commonly used criteria for autistic regression in the literature [1,2].

Participants were eligible for inclusion if they met the above-defined age range and diagnostic criteria. Children in the regression group were additionally required to meet the predefined criteria for developmental regression as described above. Typically developing controls were free of neurodevelopmental and psychiatric disorders, were not taking any medications, and showed no evidence of acute or recent infection, systemic illness, or any chronic medical conditions at the time of sample collection.

Exclusion criteria were applied to minimize potential confounding factors affecting neurodevelopment and metabolic profiles. Children were excluded if they had (i) regression onset after 36 months of age, (ii) a history of epilepsy or abnormal electroencephalographic findings, (iii) known genetic, metabolic, or neurological disorders, (iv) significant sensory impairments such as hearing loss, (v) any acute or chronic systemic illness at the time of sampling, or (vi) recent infection within the preceding two weeks. Children receiving psychotropic medications or immunomodulatory treatments were not included in the study.

The study protocol was approved by the Hacettepe University Non-Interventional Clinical Research Ethics Committee (Approval No: GO-19/1175), and written informed consent was obtained from all parents or legal guardians prior to participation. The study was conducted in accordance with the Declaration of Helsinki.

### 4.2. Sample Collection and Preparation

Venous blood samples were collected into EDTA-containing tubes during routine clinical visits. Plasma was separated by centrifugation and aliquoted into polypropylene cryovials within 30 min of collection. All aliquots were stored at −80 °C until the day of analysis. No freeze–thaw cycles occurred prior to sample preparation. A pooled quality-control (QC) sample was prepared before metabolite extraction by combining equal 20 µL aliquots from all 73 individual plasma samples (REG+, *n* = 25; REG−, *n* = 27; controls, *n* = 21). The pooled plasma was vortex-mixed for 1 min to ensure homogeneity and was subsequently processed using the same extraction procedure as the individual study samples. Equal-volume contribution from every participant ensured representation of all three study groups within the QC pool.

Metabolite extraction was performed using a single-step protein precipitation protocol. Briefly, 200 µL of plasma was transferred to a 1.5 mL Eppendorf tube and mixed with 600 µL of ice-cold LC-MS-grade methanol (plasma-to-solvent ratio of 1:3). The mixture was vortexed for 90 s. Samples were centrifuged at 9000 rpm for 10 min at 4 °C, and the clear supernatant was transferred to a fresh tube. The supernatant was dried to completeness in a vacuum centrifuge at ambient temperature. The resulting dry residue was reconstituted in mobile phase (1:1 dilution with the initial eluent composition) and transferred to autosampler vials fitted with 200 µL glass inserts for injection. The 1:3 plasma-to-methanol ratio falls within the established effective range for untargeted metabolomics protein precipitation [39].

LC-MS-grade methanol, acetonitrile, formic acid, and water were purchased from Sigma-Aldrich (St. Louis, MO, USA). All solvents and reagents were used without further purification.

### 4.3. Untargeted Metabolomics via LC-Q-TOF MS

All analyses were performed on an Agilent 6530 Accurate-Mass Q-TOF mass spectrometer coupled to an Agilent 1260 Infinity HPLC system (Agilent Technologies, Santa Clara, CA, USA). Chromatographic separation was achieved on a Zorbax Eclipse Plus C18 column (1.8 µm, 100 × 2.1 mm i.d.; Agilent), maintained at 35 °C.

The mobile phase consisted of water containing 0.1% formic acid (solvent A) and acetonitrile containing 0.1% formic acid (solvent B), delivered at 0.2 mL min^−1^. The gradient program was as follows: 10% B initially, increased linearly to 90% B over 10 min, decreased to 10% B by 17 min, and maintained at 10% B until 25 min for re-equilibration.

The mass spectrometer operated in positive ESI mode: capillary voltage 4000 V, drying gas temperature 350 °C, drying gas flow 10 L min^−1^, nebulizer pressure 45 psi, fragmentor voltage 175 V. Full-scan MS data were acquired over *m*/*z* 100–1700 with mass accuracy ≤5 ppm and resolving power >10,000 FWHM. Study samples were analyzed in randomized order. The fixed initial injection sequence and the repeating analytical block are provided in Supplementary Table S4. Six consecutive pooled QC injections were performed at the beginning of the analytical sequence for system conditioning. Thereafter, one pooled QC sample was injected after every six study-sample injections to monitor analytical stability and signal reproducibility throughout the sequence. The complete analytical sequence design, including the randomization strategy, the number and position of system-conditioning injections, the three in-house library QC injections, the mobile-phase blank and pooled QC intervals, and the analytical replication scheme, is provided in Supplementary Table S3.

### 4.4. Data Processing and Statistical Analysis

Raw data files were processed using XCMS (version 4.10.1) in R (version 4.1) [40]. Peak detection (centWave algorithm), retention time alignment, and chromatographic peak grouping generated a unified feature matrix. Features with CV >30% in QC samples were removed. Group differences were initially assessed using one-way ANOVA followed by the predefined pairwise comparisons. To account for multiple testing, raw *p*-values were adjusted separately for each comparison using the Benjamini–Hochberg false discovery rate procedure across all features retained after quality-control filtering. FDR-adjusted q-values < 0.05 were considered statistically significant, and a fold-change threshold of >1.5 was applied as an additional effect-size criterion. Raw *p*-values and corresponding FDR-adjusted q-values for the REG− vs. C, REG+ vs. C, REG+ vs. REG−, and Total ASD vs. C comparisons are reported in Supplementary Table S2. PLS-DA was used for multivariate visualization, and heat-map representations were generated.

### 4.5. Metabolite Identification and Panel Selection

Metabolite identification confidence was assigned according to the Metabolomics Standards Initiative reporting framework [41]. MSI Level 1 was assigned only to features matched against authenticated in-house reference standards using accurate mass and retention time obtained under the same analytical conditions. MSI Level 2 was assigned to putatively annotated compounds supported by accurate-mass matching (±5 ppm) against HMDB, METLIN, and KEGG, together with isotope patterns, the proposed ion/adduct form, and chromatographic elution behavior, but without confirmation using an authentic reference standard. Features for which multiple database candidates remained were explicitly designated as putatively annotated.

The initial pool of 68 annotated peaks (116 candidate assignments) was subjected to expert-curated elimination: (i) analytical plausibility (implausible adducts, retention time contradictions, >5 ppm mass error removed); (ii) endogenous origin (pharmaceutical agents, plant sterols, bacterial alarmones excluded); (iii) isomeric resolvability (unresolvable isomer sets downgraded). This reduced the dataset to 32 features.

A final selection step identified the 14-metabolite panel (Table 1) among features that remained significant after Benjamini–Hochberg correction (q < 0.05) and met the fold-change threshold of >1.5 in at least one comparison, followed by assessment of analytical plausibility, endogenous origin, and biological relevance. Pathway enrichment analysis was performed in MetaboAnalyst 5.0 [42]. The panel was organized post hoc into four biological narrative axes for interpretive clarity; this grouping did not influence statistical analysis.

Detailed analytical and statistical information for the 68 unique chromatographic features and 116 candidate metabolite assignments, including observed *m*/*z*, retention time, proposed annotation, KEGG and HMDB identifiers, ion/adduct form, mass error, fold-change values, direction of change, raw *p*-values, Benjamini–Hochberg FDR-adjusted q-values, MSI identification level, and the basis of identification, is provided in the Supplementary Table S2.

## 5. Conclusions

This exploratory study identified a set of plasma metabolic features showing different abundance patterns among children with REG+, REG−, and typical development. A feature putatively annotated as PGE3 showed markedly lower abundance in the regressive ASD group, while sialylated glycoconjugates and glycoprotein turnover intermediates showed comparatively larger alterations in this subgroup. These observations are consistent with possible differences in inflammation-related and N-linked glycan pathways, superimposed on sphingolipid–myelin alterations observed across both ASD groups. However, the metabolite annotations and the proposed biological interpretations require confirmation using authenticated reference standards and targeted quantitative methods.

The observed two-tier pattern—shared sphingolipid–myelin alterations together with more pronounced inflammation- and glycosylation-related differences in the regressive subgroup—provides a hypothesis for future longitudinal investigation. Because ROC/AUC analysis and independent validation were not performed, the present findings do not establish diagnostic sensitivity, specificity, predictive value, or pharmacodynamic utility. Replication in larger, independently recruited cohorts will be required before the clinical relevance of these metabolic features can be determined.

## Figures and Tables

**Table 1:** Differential plasma metabolic features showing abundance differences among children with REG+, REG−, and typically developing controls (C). Metabolites are grouped by biological pathway narrative. Only metabolic features meeting an FDR-adjusted significance threshold of q < 0.05 and a fold-change threshold of >1.5 in at least one comparison, and passing the analytical and biological plausibility filters, are included.

Metabolite (Class)	Biological Pathway	FC (REG+/C)	FC (REG−/C)	FC (REG+/REG−)	Trend (REG+)
Sphingolipid–Myelin Axis					
Sphinganine 1-phosphate (Sphingoid base phosphate)	De novo sphingolipid signaling; BBB regulation	2.40 ↑	2.36 ↑	1.02	↑
Sphinganine (Sphingoid base)	De novo sphingolipid biosynthesis	1.54 ↓	1.73 ↓	1.13	↓
Dihydroceramide (Ceramide precursor)	Myelin lipid biosynthesis; membrane integrity	4.16 ↓	5.22 ↓	1.25	↓↓
Galactosylceramide (d18:1/16:0) (Glycosphingolipid)	Principal myelin glycolipid	3.53 ↓	3.91 ↓	1.11	↓↓
Palmitaldehyde (Fatty aldehyde)	Terminal product of sphingolipid catabolism	1.70 ↑	1.76 ↑	1.04	↑
Ganglioside GA2 (d18:1/12:0) (Glycosphingolipid)	Synaptic ganglioside biosynthesis	2.00 ↓	2.13 ↓	1.06	↓
Inflammation Resolution Pathway					
Prostaglandin E3 (Eicosanoid)	EPA-derived anti- inflammatory prostaglandin	7.13 ↓	1.58 ↓	4.52 ↓	↓↓↓
LysoPC (18:1(9Z)) (Lysophospholipid)	PLA_2_ product; arachidonate release marker	1.26 ↑	1.53 ↑	1.21	↑
Glycosylation and Sialylation Axis					
N-Acetylneuraminosyl-(α2-6)lactosamine (Sialylated trisaccharide)	Cell surface sialylation; NCAM function	4.44 ↓	1.83 ↓	2.43 ↓	↓↓↓
Aspartylglycosamine (Glycoamino acid)	N-linked glycoprotein turnover	5.42 ↓	1.75 ↓	3.09 ↓	↓↓↓
Gut–Brain Axis and Systemic Metabolism					
Deoxycholic acid (Secondary bile acid)	Gut microbiota bile acid transformation	6.97 ↑	5.84 ↑	1.19	↑↑↑
L-Tryptophan (Essential amino acid)	Serotonin–kynurenine precursor	1.35 ↓	1.76 ↓	1.31	↓
Cholesterol (Sterol)	Membrane biosynthesis; synaptogenesis	2.37 ↓	1.73 ↓	1.37	↓↓
DHEA-S (Neurosteroid sulfate)	Adrenal neurosteroid; HPA axis regulation	1.94 ↓	17.23 ↓	8.89 ↑	↓

Abbreviations: FC, fold change; REG+, children with regressive ASD (*n* = 25); REG−, children with non-regressive ASD (*n* = 27); C, typically developing healthy controls (*n* = 21); DHEA-S, dehydroepiandrosterone sulfate; EPA, eicosapentaenoic acid; PLA_2_, phospholipase A_2_; NCAM, neural cell adhesion molecule; BBB, blood–brain barrier; HPA, hypothalamic–pituitary–adrenal. FC interpretation: FC (REG+/C) denotes the fold change between the regressive ASD group and controls, reflecting the magnitude of metabolic alteration in children who have experienced developmental regression. FC (REG−/C) denotes the fold change between the REG− group and controls, capturing metabolic alterations shared across the broader autism spectrum irrespective of regression history. FC (REG+/REG−) describes the numerical fold-change difference between the REG+ and REG− groups. Larger values indicate greater subgroup-associated abundance differences but should not be interpreted as evidence of diagnostic discrimination, sensitivity, specificity, or predictive performance. Trend arrows: ↑ indicates an increase and ↓ indicates a decrease relative to the control group. The number of arrows reflects the approximate magnitude (↓ < 2-fold; ↓↓, 2–5-fold; ↓↓↓, > 5-fold decrease; and equivalently for increases). Pathway grouping: Metabolites are organized by biological narrative rather than chemical class to facilitate interpretation. Several metabolites participate in more than one pathway (e.g., ganglioside GA2 bridges the sphingolipid–myelin and glycosylation axes); placement reflects the primary narrative relevance. Analytical note: All features were detected by untargeted Q-TOF LC/MS (Agilent 6530) in ESI+ mode with a mass accuracy of ≤5 ppm. Identification confidence corresponds to MSI Level 1 or MSI Level 2, as specified individually in Table S2. MSI Level 1 identifications were established by matching accurate mass and retention time against authenticated in-house reference standards analyzed under the same conditions. MSI Level 2 identifications represent putative annotations supported by accurate mass, isotope pattern, chromatographic behavior, and database matching, without confirmation using an authentic reference standard.

## Data Availability

The processed feature-level metabolomics results supporting the findings of this study are provided in the Supplementary Table S1. The table includes observed *m*/*z* and retention time values, proposed metabolite annotations, database identifiers, ion/adduct information, mass errors, raw *p*-values and Benjamini–Hochberg FDR-adjusted q-values for the REG− vs. C, REG+ vs. C, REG+ vs. REG−, and Total ASD vs. C comparisons, fold-change values, direction of change, MSI identification level, and the basis of identification for 68 unique chromatographic features and 116 candidate assignments. The analytical sequence design and quality-control scheme, including sample randomization, system conditioning, pooled QC and mobile-phase blank injection intervals, and analytical replication, are provided in Supplementary Table S3. The vendor-specific raw LC–QTOF-MS data files and associated analytical files are available from the corresponding author upon reasonable request due to privacy and ethical restrictions related to human participant data.

## Supplementary Materials

The following supporting information can be downloaded at: https://www.mdpi.com/article/10.3390/ijms27156995/s1.
